# TrialTriage, a Semiautonomous Prescreening Workflow for Resolving Ambiguity in Phase I Oncology Trial Eligibility: Development and Proof-of-Concept Study Using Synthetic Cases

**DOI:** 10.2196/100779

**Published:** 2026-08-05

**Authors:** Kenneth A Kern

**Affiliations:** 1 Affiliate Faculty, Skaggs School of Pharmacy and Pharmaceutical Sciences, University of California San Diego, La Jolla, CA, United States; 2 Oncology Breakthrough Advisory Group, LLC, 1804 Garnet Avenue, Suite 701, San Diego, CA, 92109, United States, 1 (619) 354-1026

**Keywords:** clinical trials, prescreening, phase I, oncology, patient eligibility, clinical trial matching, workflow automation, large language model, LLM, natural language processing, semiautonomous AI, feasibility study, patient accrual

## Abstract

**Background:**

Enrollment in phase I oncology trials remains low largely because potentially eligible patients are not identified and evaluated quickly enough. Current clinical trial matching systems can identify candidate patients from the electronic health record, but cases with missing or uncertain eligibility data are often routed for offline manual review. This delay impedes clarification and prolongs the final eligibility determination.

**Objective:**

This study evaluated TrialTriage, a semiautonomous system built on the n8n platform and designed to resolve eligibility ambiguity during prescreening for phase I oncology trials. When eligibility information is missing or uncertain, TrialTriage emails the investigator, captures the reply, and reruns classification within the same workflow.

**Methods:**

TrialTriage combined large language model–based variable extraction from free-text clinical narratives and investigator email replies with a deterministic rule engine applying a prespecified 7-criterion protocol. Each case was classified as eligible, not eligible, or ambiguous. Ambiguous cases triggered a structured email query to the investigator, followed by reclassification after a reply. Two requests were sent at 24-hour intervals; after 48 hours without a reply, the case was referred for manual review. The system was tested on 90 synthetic patient cases generated independently by Claude Sonnet 4.6, Gemini 3.1, and Grok 4, with 30 cases per model and balanced distributions of eligible, not eligible, and ambiguous cases. Answer keys were reviewed for accuracy before system execution. Five independent reviewers classified the Claude dataset using a uniform survey form.

**Results:**

TrialTriage’s classifications were 100% concordant with the author-confirmed ground truth in all 90 synthetic cases (95% CI 96.0%-100.0%). All ambiguous cases were correctly escalated to investigator query. The mean processing time was 2.3 (SD 0.5) minutes per 30-case dataset (range 1.8-2.8 min, approximately 3.5-5.5 s per case). The 5 reviewers achieved a mean accuracy of 96.7% (SD 3.3%), with a Fleiss κ of 0.910, and required a mean of 9.8 (SD 4.8) minutes to review 30 cases. In a subset test of 6 first-pass ambiguous cases, 4 of 6 were reclassified definitively after investigator response, while 2 remained ambiguous because the replies lacked actionable information.

**Conclusions:**

TrialTriage demonstrates the feasibility of a semiautonomous prescreening workflow in which ambiguous cases trigger an immediate investigator email query and are reclassified after reply capture with new information within the same system. The main contribution is the integration of email ambiguity resolution into the workflow rather than immediate deferral to offline manual review. Because the evaluation used synthetic cases and label definitions aligned with the same protocol rules used to design the rule engine, these findings should be interpreted as proof of concept and implementation fidelity rather than evidence of real-world clinical performance. Prospective validation using data from real-world electronic health records would be a plausible next step.

## Introduction

Phase I oncology trials are the initial clinical testing platform for new anticancer drugs, either alone or in combination [1-3]. These trials establish safety, tolerability, and preliminary dosing. Their successful completion is a prerequisite for progression to later-phase trials that support regulatory approval [4]. Robust interpretation of phase I data requires the enrollment of a predefined sample size [4,5]. Reaching that sample size depends on identifying and enrolling patients who meet trial entry criteria through a 2-step process known as eligibility screening.

Eligibility screening for phase I oncology trials is conducted in 2 sequential steps: prescreening followed by formal screening [5,6]. Prescreening is an initial review of the electronic health record (EHR) or oncologist’s patient listing against a limited set of key trial entry criteria, conducted without patient contact. Patients identified as potential candidates are then contacted, the trial is explained, and those who express interest are asked to sign an informed consent document to allow formal screening. Formal screening establishes final eligibility through detailed disease assessment, including physical examination, laboratory testing of major organ function, and radiographic evaluation of disease extent. Patients who satisfy all criteria after formal screening are enrolled [7,8]. Both steps are challenging in phase I oncology because of strict entry criteria, the unpredictable safety profile of novel agents, and the narrow patient populations these trials require.

Fewer than 8% of eligible patients with cancer enroll in clinical trials [9-11]. This low accrual rate is not primarily driven by patient refusal: between 55% and 80% of patients identified as eligible and invited agreed to enroll [12-14]. The dominant barrier is the inefficient identification of the smaller subset of patients who meet entry criteria from among many patients who do not [6,15]. Among patients who are prescreened and reach formal screening, approximately 25% fail to meet eligibility criteria and are termed “screen failures” [16,17]. Because formal screening is expensive and resource-intensive, this rate raises the question of whether better prescreening could improve the screening success rate and increase trial enrollment.

Improving low enrollment rates would have significant consequences for early-phase trial completion. Many phase I studies terminate early due to low enrollment rates and failure to meet statistically defined sample sizes. In one analysis of terminated trials, 57% closed specifically because of insufficient accrual of patients rather than drug toxicity or lack of antitumor activity [10].

Phase I oncology trials typically last 2 to 3 years, with low enrollment rates as a primary contributor to delays [5,18]. For patients with malignant disease, these delays extend the time required for potentially effective therapies to reach them [19].

Prescreening is typically performed manually by clinical research staff, who review records, query databases for missing information, and escalate uncertain cases to clinicians. These steps are labor-intensive, and a substantial fraction of patients cannot be definitively classified at this stage. Prior studies of clinical trial matching (CTM) systems report that 20% to 25% of prescreened patients cannot be definitively classified because of incomplete or ambiguous data and require manual adjudication [20,21]. High model performance does not resolve this problem on its own. A prospective randomized trial of 20,707 patients showed no enrollment impact despite high model performance (area under the receiver operating characteristic curve=0.91), largely because 23.2% of notifications were rejected due to a lack of clinical factors key to protocol entry [22]. Delay in resolving ambiguity has direct clinical consequences: processing delays of 48 hours after imaging detects the progression of disease have resulted in 20% of patients starting alternative treatments before trial notification occurs [23]. Although multiple factors contribute to accrual delays, unresolved eligibility ambiguity at the prescreening stage is a key driver of enrollment inefficiency and a documented contributor to the screen-failure rate described above [16,17,24].

AI approaches have been developed to support patient identification through the analysis of structured and unstructured clinical data [25,26]. These systems, often described as CTM systems, analyze eligibility criteria and classify patients as eligible, not eligible, or ambiguous. In current CTM systems, cases classified as ambiguous are typically removed from the automated workflow and sent for manual review; information obtained during that review is generally not returned to the original classification process [27]. As a result, ambiguity is not resolved within the workflow itself but is shifted into a slower manual process, increasing delays and creating additional opportunities for inconsistency or error.

To address this limitation, we designed TrialTriage, a semiautonomous workflow for prescreening in phase I oncology trials. TrialTriage classifies patients as eligible, not eligible, or ambiguous and automatically sends an email to the investigator when required information is missing or unclear. The system’s large language model (LLM) evaluates the investigator’s reply, extracts any substantive clarifying information, and passes that information to the rule engine for reclassification within the same workflow. This design builds on the broader principle that corrective human input can improve classification accuracy, as reported in a study in which manual feedback increased performance from 88.0% to 92.7% [20]. TrialTriage extends that principle by automatically requesting feedback and clarification of ambiguous cases by email within the workflow, rather than leaving those cases for manual review outside of it.

The aim of this study was to develop and evaluate TrialTriage, a semiautonomous prescreening workflow for phase I oncology trials, whose novel component is an immediate, iterative email exchange with the principal investigator (PI). There were 2 key objectives for this study. The first was to determine whether TrialTriage could perform the following functions: correctly classify synthetic phase I oncology cases as eligible, not eligible, or ambiguous; route a listing of ambiguous cases and the data needed to classify them to an investigator through immediate email query; apply an iterative process that uses PI responses to reclassify ambiguous cases to the correct eligibility category; and retain unresolved cases as ambiguous for manual classification. The second was to compare TrialTriage’s classification accuracy and processing time against those of human reviewers evaluating the same cases.

We hypothesized that TrialTriage would classify cases in complete agreement with the predefined ground truth and do so in less time than human reviewers, even when those reviewers were experienced in phase I trials, and that TrialTriage would request additional data from the PI and reclassify ambiguous cases accurately. Because the evaluation used synthetic cases built from the same criteria used to design the rule engine, we framed these as tests of internal validity and implementation fidelity, not of real-world clinical performance. This approach aligns with calls to reduce screen failure through automation [28] and with current regulatory guidance emphasizing transparent, auditable outputs and structured human oversight for AI used in clinical trials [29,30].

## Methods

### Study Design

This proof-of-concept study evaluated a semiautonomous workflow for prescreening synthetic patient cases against the eligibility criteria of a hypothetical phase I oncology trial. Synthetic cases were generated by LLMs to simulate patients with advanced pancreatic cancer, as described below. Pancreatic cancer was used as a representative disease to test eligibility in a hypothetical phase I oncology trial. The architecture of the workflow is not specific to any one tumor type and could be generalized to other phase I oncology trials.

### Ethical Considerations

This study did not involve human subjects and did not use actual patient data. All clinical cases were synthetic narratives generated by LLMs. Because this study involved only synthetic patient data and no living individuals underwent data collection, it did not constitute human subjects research and did not require institutional review board review or approval under the US Common Rule (45 CFR 46.102). The 5 clinicians who classified cases for the manual comparison did so as expert colleagues evaluating synthetic cases as part of a methods comparison. Since they were not research subjects, no personal data about these reviewers were recorded beyond their professional role—physician or nonphysician clinician—and the time taken to complete the classification task.

### System Architecture

**Figure 1.**
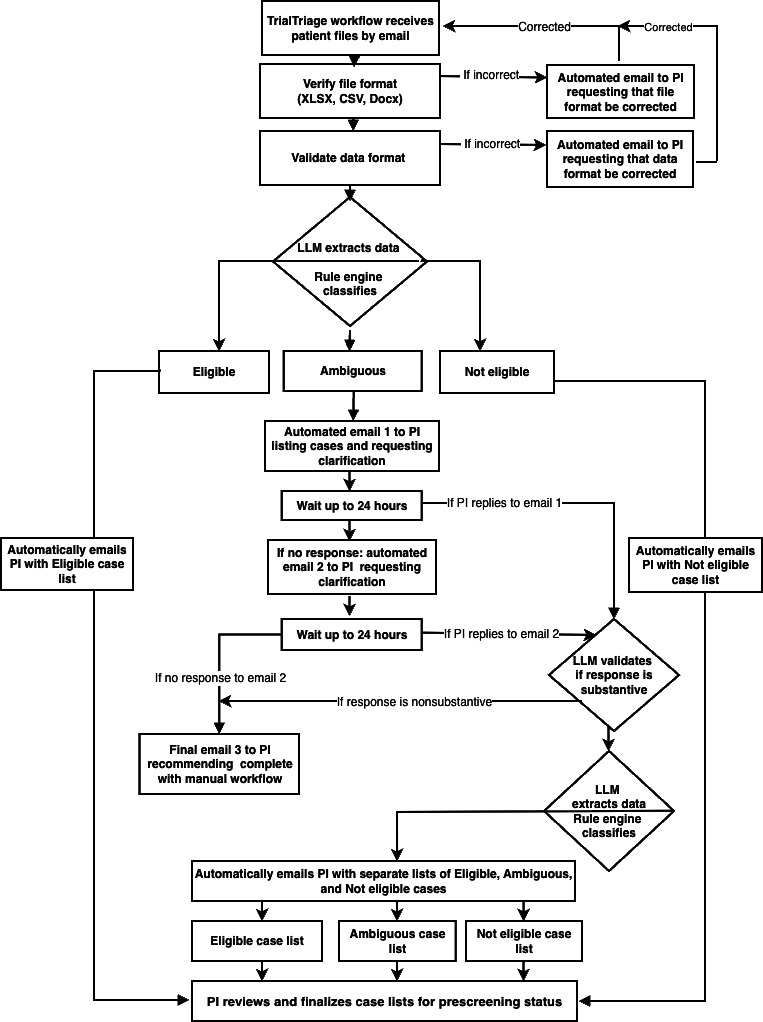
Simplified TrialTriage architecture. Patient files are received by email and validated. The large language model (LLM) extracts the 7 eligibility variables; a deterministic rule engine classifies each case as eligible, ambiguous, or not eligible. Ambiguous cases trigger an automated email to the principal investigator (PI), with one escalation if no reply is received within 24 hours. Substantive replies are reextracted by the LLM and reclassified by the rule engine. Unresolved cases exit to a recommended manual workflow. Diamond shapes indicate decision steps.

TrialTriage is a hybrid architecture orchestrated on the n8n workflow automation platform (n8n GmbH). In n8n, individual workflow steps, called “nodes,” are connected through a visual interface to define a sequence of operations. The TrialTriage workflow comprises 3 functional components: intake and preprocessing, classification and iterative reclassification, and communication and output. These components are organized into 5 sequential stages: input processing, initial classification, first investigator email, ingestion of additional data for ambiguous cases, and final case reclassification and output. A simplified architecture for the TrialTriage workflow is shown in Figure 1.

The 5 stages are implemented using 45 deployed node instances drawn from 20 unique node types. Three of these node types incorporate LLMs: an Extract from File node, which extracts the 7 eligibility variables from free-text input; a Text Classifier node, which categorizes investigator replies as substantive or nonsubstantive; and an AI Agent node, which converts substantive reply text into structured data for reingestion by the rule engine. All 3 node types use Anthropic’s Claude Sonnet 4.6 as the underlying language model. References to “Agent” elsewhere in this *Methods* section refer specifically to the n8n AI Agent node type.

Claude Sonnet 4.6 was selected as the model embedded within the 3 LLM-based nodes for 2 reasons. First, Claude was already integrated as a selectable model within the n8n platform, which simplified deployment and avoided the need for custom API handling. Second, the rule engine had been developed using GPT-5 (OpenAI) during Version 1 of TrialTriage, and choosing a different LLM for Version 2 provided some independence from that earlier development.

Screenshots of the workflow architecture are provided in Multimedia Appendix 1. A complete listing of the n8n node types used in the workflow, along with their deployed instance counts, is provided in Multimedia Appendix 2. The version of n8n used in this study retained timestamped records of each classification, email query, response ingestion, and reclassification event for up to 7 days, creating an audit trail of workflow activity. An enterprise version of n8n, not used in this report, can maintain longer-term storage.

### Data Ingestion, Validation, and Initial Classification

When a patient file is received by email, an input validation node checks both the file format and data format. Accepted file formats are XLSX, CSV, and DOCX. If either check fails, the system sends an automated email to the PI identifying the format problem and requesting resubmission. Only files and data that pass validation proceed to downstream processing. After format validation, the system’s LLM, Claude Sonnet 4.6 (Anthropic), extracts the patient ID and eligibility variables from the free-text clinical narrative for each patient. A deterministic rule engine then assigns each case to one of 3 categories: eligible, not eligible, or ambiguous.

### Eligibility Criteria

Seven eligibility criteria, representative of those commonly used in prescreening patients with pancreatic cancer for a phase I oncology trial, were defined for this study (Table 1). Thresholds were approximate and were not intended to reproduce any specific existing trial. The system was designed to accept “pancreatic cancer” as synonymous with “pancreatic adenocarcinoma” to avoid misclassification based on terminology alone. Cases were classified as eligible only if all 7 criteria were documented in the case narrative as meeting entry requirements.

**Table 1.** The 7 eligibility criteria used by the TrialTriage rule engine to classify synthetic phase I oncology trial cases.

Criterion	Threshold
Histology	Pancreatic adenocarcinoma (pancreatic cancer) confirmed by pathology within the previous 6 months
Age	18‐80 years, inclusive
ECOG^a^ performance status	0 or 1: ECOG functional scale, 0‐4, where 0 indicates fully active and 4 indicates completely disabled and confined to bed or chair
AST^b^ (SGOT^c^)	≤40 U/L
ALT^d^ (SGPT^e^)	≤40 U/L
Total bilirubin	≤1.2 mg/dL
Creatinine	≤1.5 mg/dL

a ECOG: Eastern Cooperative Oncology Group.

b AST: aspartate aminotransferase.

c SGOT: serum glutamic-oxaloacetic transaminase.

d ALT: alanine aminotransferase.

e SGPT: serum glutamic-pyruvic transaminase.

### Classification Categories

After the system extracts the eligibility variables and the rule engine classifies each case, the system groups the results by category and sends 3 separate emails to the PI: one listing all eligible cases, one listing all not eligible cases, and one listing all ambiguous cases. When a case is classified as ambiguous, the system sends the investigator a structured email requesting the specific missing or unclear information needed for reclassification. When a substantive response is received, the workflow ingests the investigator’s reply and reruns extraction and classification to generate an updated classification. After reclassification, the system again sends 3 category emails to the PI containing the updated eligible, not eligible, and remaining ambiguous cases. A final email asks the PI to review and approve all classifications.

### Closed-Loop Workflow Execution and Final Eligibility Decisions

The workflow executes a closed-loop sequence of data ingestion, classification, action, and reingestion without human intervention between steps. Specifically, it extracts narrative information, classifies cases through the rule engine, sends emails and schedules reminders, receives investigator responses, and reclassifies cases when new information is provided. The architecture is read-only with respect to any upstream clinical data source: the workflow does not write back to the EHR or modify original input data [30]. As shown in Figure 1, the workflow outputs case lists for eligible, not eligible, and ambiguous categories after both the initial classification and any iterative reclassification. Final eligibility decisions for all cases remain the responsibility of the PI.

### Synthetic Dataset Generation

TrialTriage was built in 2 versions: Version 1, a limited prototype; and Version 2, the system presented in this report. Both versions used the same 7 eligibility criteria. In both versions, synthetic cases were generated using 4 LLMs, each prompted independently: ChatGPT-5.0 (OpenAI), Claude Sonnet 4.6 (Anthropic), Gemini-3.1 (Google), and Grok 4 (xAI). The same prompt structure was used across models (Multimedia Appendix 3). Each model was instructed to generate brief but realistic synthetic case narratives of patients with pancreatic cancer, 2 to 3 sentences in length, using the 7 protocol-specific eligibility criteria described above.

Version 1, built and tested using 50 ChatGPT-generated cases, performed initial classification only. It produced eligible, not eligible, or ambiguous category emails and did not include an iterative investigator email loop or reclassification. Version 1 is not reported here, and the Version 1 synthetic cases are not included in the multimedia appendix. In Version 2 of TrialTriage, evaluated in this report, additional functions included an iterative investigator email loop, reclassification of ambiguous cases, and a substantiveness check on investigator replies.

Version 2 of TrialTriage was evaluated on cases generated independently by Claude Sonnet 4.6, Gemini-3.1, and Grok 4. Each of the 3 models generated 30 clinical scenarios in randomized order, comprising 10 eligible cases, 10 not eligible cases, and 10 ambiguous cases. Each model also produced an answer key specifying the correct classification for each case. The 90 cases were all different clinical scenarios, produced using different permutations of the 7 eligibility criteria, with no case appearing in more than 1 model’s dataset. The synthetic case datasets were generated in February 2026. All answer keys were checked by the author to confirm the ground truth. The full case listings, with ground-truth labels and classification outputs, are provided in Multimedia Appendix 4.

### Evaluation Procedure

The workflow included a built-in evaluation branch that permitted either a regular workflow mode, triggered by a Gmail message with a specified subject line, or a separate evaluation mode run from spreadsheet tabs stored in Google Sheets. Initial testing used the 30 Claude-generated cases. The Gemini and Grok datasets were then submitted to the system to assess concordance with independently generated inputs. TrialTriage classification of all 90 cases was performed in March 2026. TrialTriage classifications for each case are included alongside the case listings in Multimedia Appendix 4.

### Ground Truth

Each model generated an answer key specifying the correct classification for each case. During the Grok evaluation, the author identified disagreements with the model-generated answer keys in more than 50% of cases. Investigation determined that the rule engine recognized only “pancreatic adenocarcinoma,” whereas some Grok-generated cases used the medically synonymous term “pancreatic cancer.” Both terms refer identically to cancer of the pancreas. The rule engine, which is deterministic and not a trained model, was modified to accept “pancreatic cancer” as equivalent. The final evaluation of all 90 cases was conducted on the modified rule engine. Classifications agreed with the author-confirmed ground truth in all 90 cases.

Because Claude served both as one of the 3 case generators and as the embedded LLM in the current TrialTriage, we checked whether TrialTriage favored cases produced by Claude by comparing its performance on cases it generated versus those generated by Gemini and Grok. Following the correction of the pancreatic cancer definition described above, classification was 100% concordant across the Claude, Gemini, and Grok cases. This single-pass evaluation was not designed to measure run-to-run variability, which remains untested and is noted as a limitation.

### Investigator Clarification for Ambiguous Cases

Cases classified as ambiguous triggered immediate email communication, with the PI requesting clarification of the specific missing or uncertain data elements. The first email listed each ambiguous case by ID along with the relevant clinical narrative and contained a “Respond” button. When clicked, the button opened a reply email prompting the PI to provide the specific missing or unclear data for each identified case. If no reply was received within 24 hours, a second email was sent requesting a response. If no reply was received within an additional 24 hours, a third and final email informed the PI (or, in future designs, other designated research staff) that the case was being removed from the automated classification system and needed to be reviewed through a manual process. The workflow then took no further action on classifying the case and relied on the PI to have the case evaluated by the site’s standard manual prescreening process.

Routing the case directly from the workflow to a named secondary reviewer or coordinator was not implemented in this study and is identified in the *Future Directions* section as a planned enhancement. Investigator replies were evaluated by the LLM and classified as substantive or nonsubstantive. Substantive replies contained actionable clinical information relevant to one or more ambiguous eligibility criteria. Nonsubstantive replies lacked actionable clinical information, such as “I do not have that information,” “I will investigate it,” or out-of-office replies.

### Comparison of Manual Prescreening Performance to TrialTriage

Five reviewers manually classified the randomized 30-case Claude dataset using the same 7 eligibility criteria applied by TrialTriage. Reviewers were blinded to the answer key. Three reviewers were physicians with extensive drug development experience, while the remaining 2 were nonphysician clinicians with similarly extensive drug development experience. None had seen the cases prior to evaluation. The 5 reviewers completed the manual classification of the Claude dataset between March 4 and March 30, 2026. Each reviewer independently recorded classifications for all 30 cases on a separate survey sheet (Multimedia Appendix 5), assigning each case as eligible, not eligible, or ambiguous. At the top of each survey sheet, before the case listings, instructions defined the 3 classifications and stated that any case with missing, pending, vague, or nonnumeric data should be classified as ambiguous. Average classification time per case was calculated by dividing each reviewer’s total classification time by 30. Manual review was limited to the Claude dataset; performance against the Gemini and Grok datasets was not assessed.

### Statistical Analysis

Classification accuracy was reported with exact binomial 95% CIs. Agreement between each reviewer and the ground truth was quantified using Cohen κ with 95% CI. Interrater agreement across all 5 reviewers was quantified using Fleiss κ. The 95% CI was estimated by nonparametric bootstrap resampling over 5000 iterations, drawing from the table of how each reviewer classified each case. This was a post hoc analysis performed in Python 3.12.3 (Python Software Foundation) with NumPy 2.4.4 (NumFOCUS, Inc), outside the n8n workflow.

Total time required to classify the 30-case dataset was summarized descriptively. Reviewer times were reported as the mean (SD) across the 5 reviewers, while TrialTriage times were reported as the observed range across the 30 cases. TrialTriage and reviewer classification times were compared descriptively rather than inferentially because both sample sizes were small. This paper follows the TRIPOD-LLM reporting guideline, which specifies what studies that develop or evaluate LLMs in health care should report [31]. A completed checklist is provided in Checklist 1.

## Results

### Datasets and Evaluation

TrialTriage was evaluated on the 90 synthetic cases described in *Methods*, comprising three 30-case datasets generated by Claude Sonnet 4.6, Gemini-3.1, and Grok 4. The Claude dataset served as the primary evaluation set. TrialTriage was applied to all 3 datasets, but the iterative investigator email clarification loop was applied only to the Claude dataset. The 5 reviewers also classified only the Claude dataset. The Gemini and Grok datasets were used to test the consistency of TrialTriage’s performance across cases generated by different LLMs. Complete case narratives, ground-truth labels, TrialTriage classifications, and reviewer classifications are provided in Multimedia Appendix 4.

### TrialTriage Classification Performance

TrialTriage’s classifications were concordant with the author-confirmed ground truth in all 90 cases across the 3 evaluation datasets (90/90; exact binomial 95% CI 96.0%-100.0%), including all eligible, not eligible, and ambiguous cases. Concordance was 30/30 on the Claude dataset, 30/30 on the Gemini dataset, and 30/30 on the Grok dataset. Classification remained stable across datasets generated by different LLMs, despite differences in wording and case presentation. Because the synthetic cases were generated by LLMs prompted with the same 7 eligibility criteria from which the rule engine was constructed, this finding represents internal consistency of the workflow on prompt-faithful narratives rather than performance on the real clinical text.

### System Processing Time

TrialTriage processed each 30-case dataset in 1.8 to 2.8 minutes, corresponding to 3.5 to 5.5 seconds per case. Total processing time across all 90 cases was 5.4 to 8.4 minutes. This timing included data ingestion, LLM extraction of structured variables, and rule-engine classification. Per-dataset processing times for the Claude, Gemini, and Grok datasets all fell within this range. All 90 cases were processed without the interruption of the n8n workflow. These figures reflect automated processing time only and do not include the 24-hour escalation intervals or investigator response time required for ambiguous cases entering the email clarification loop.

### Iterative Reclassification of Ambiguous Cases

The iterative investigator feedback loop was evaluated on 6 of the 10 ambiguous cases in the Claude primary dataset, selected at random. The author served as a simulated PI and replied to the workflow’s automated email queries; this single-responder design is acknowledged in the *Limitations* section. For 4 cases, the author provided additional clarifying data involving ECOG (Eastern Cooperative Oncology Group) status, confirmation of diagnosis, or laboratory values within the range. The rule engine reclassified all 4 as eligible after the LLM re-extracted variables from the reply, consistent with the data the author provided. For the remaining 2 cases, the author responded with nonsubstantive statements such as “data not available” or “I don’t have that information.” Both cases were retained as ambiguous cases and triggered the workflow’s recommendation for manual classification, as specified in the *Methods* section. All replies in this test were submitted within 24 hours, so the second-email escalation path was not triggered or evaluated.

### Manual Reviewer Performance

Reviewer accuracy on the 30-case Claude dataset ranged from 93.3% to 100.0%, with a mean of 96.7% (SD 3.3%); per-reviewer accuracy, classification time, and Cohen κ are shown in Table 2. Interrater agreement across all 5 reviewers was almost perfect according to the Landis and Koch benchmarks [32] (Fleiss κ=0.910; bootstrap 95% CI 0.813-0.980). The mean reviewer classification time was 9.8 (SD 4.8) minutes per 30 cases, about 20 seconds per case. TrialTriage classified the same 30 cases in 1.8 to 2.8 minutes, about 4 to 6 seconds per case, with no errors. TrialTriage was therefore about 4 times faster.

**Table 2.** Reviewer and TrialTriage performance on the 30-case Claude dataset.

Reviewer	Role	Time (min)	Time (s)	Correct	Accuracy (%)	Cohen κ^a^ (95% CI)
Reviewer A	MD^b^	6.0	360	30/30	100.0	1.000^c^
Reviewer B	MD	18.0	1080	30/30	100.0	1.000^c^
Reviewer C	MD	6.9	415	29/30	96.7	0.950 (0.854‐1.000)
Reviewer D	Non-MD	10.0	600	28/30	93.3	0.900 (0.766‐1.000)
Reviewer E	Non-MD	8.0	480	28/30	93.3	0.900 (0.766‐1.000)
Mean (SD)	—^d^	9.8 (4.8)	587 (290)	—	96.7 (3.3)	—
TrialTriage	—	1.8‐2.8	105‐165	30/30	100.0	1.000^c^

a Cohen κ is reported for each reviewer relative to the ground-truth answer key, with asymptotic 95% CI. Interrater agreement across all 5 reviewers: Fleiss κ=0.910 (bootstrap 95% CI 0.813-0.980, 5000 resamples).

b MD: physician.

c The CI is not calculable when κ=1.000, with no observed disagreement.

d Not applicable.

### Manual Reviewer Errors

Five misclassifications occurred across the 150 reviewer classifications; each is listed by case, reviewer, and failure mode in Table 3. Four of the 5 errors reflected overinclusion of patients who did not meet the criteria or the premature resolution of ambiguity, and no reviewer classified an eligible case as not eligible or ambiguous. TrialTriage made no classification errors on the same dataset.

**Table 3.** Manual reviewer classification errors in the 30-case Claude dataset^a^.

Case ID	Reviewer	Correct class	Reviewer response	Error	Failure mode
5	C	Not eligible	Eligible	Not eligible→Eligible	Age of 83 years exceeds the upper limit of 80 years; should be classified as not eligible
5	D	Not eligible	Eligible	Not eligible→Eligible	Age of 83 years exceeds the upper limit of 80 years; should be classified as not eligible
6	E	Ambiguous	Not eligible	Ambiguous→Not eligible	Bilirubin absent from the lab panel; missing data should be classified as ambiguous
16	D	Ambiguous	Eligible	Ambiguous→Eligible	No ECOG^b^ numeric status, narrative only; should be classified as ambiguous
18	E	Not eligible	Eligible	Not eligible→Eligible	SGPT^c^ 57 U/L exceeds ULN^d^ of 40 U/L; should be classified as not eligible

a Error column shows the misclassification as correct class→reviewer response.

b ECOG: Eastern Cooperative Oncology Group.

c SGPT: serum glutamic-pyruvic transaminase.

d ULN: upper limit of normal.

## Discussion

### Principal Findings

TrialTriage met both objectives of this proof-of-concept evaluation. The first objective was to determine whether TrialTriage could classify synthetic phase I oncology cases, route ambiguous cases to the investigator, reclassify them based on the PI’s response, and retain unresolved cases for manual classification. TrialTriage successfully classified every synthetic case in agreement with the predefined ground truth across datasets generated by 3 different LLMs, categorizing each as eligible, not eligible, or ambiguous. It routed every ambiguous case through an immediate, structured email query, reclassified the cases that received substantive replies, and retained the remaining cases as Ambiguous for manual classification. The second objective was to compare TrialTriage’s classification accuracy and processing time against those of human reviewers evaluating the same cases. TrialTriage classified cases faster than the reviewers and without classification errors, whereas the reviewers’ errors involved either overinclusion or premature resolution of ambiguous cases.

### Comparison With Prior Work

Existing CTM systems and TrialTriage can be compared based on 2 points: what they do with the cases they cannot classify and how accurately they classify. Current systems focus their automated workflow primarily on identifying eligible patients, and they divert ambiguous cases for offline, manual resolution, which is often delayed [33-35]. When clinical observations are the only source for an eligibility variable, or when variables related to the past medical history are absent or unclear, automated CTMs cannot resolve the case definitively, and the case is routed for manual review. Manual review of such cases is time-consuming and resource-intensive [6]. TrialTriage operates downstream of these systems, prescreening identified candidates against a specific trial’s criteria and resolving ambiguity within the same workflow.

We surveyed published descriptions of CTM and prescreening systems, including Tempus Tapp [36], TrialMatchAI [37], OncoLLM [38], Watson CTM [21], TrialGPT [39], and a natural language processing–based screen failure prediction model [19]. None of these systems describe an automated mechanism for resolving ambiguous cases within the workflow. Several do not address ambiguous cases at all, leaving it unclear whether such cases are excluded from analysis, defaulted to one classification, or routed for offline manual review.

In a cohort of 102 patients with non–small cell lung cancer, IBM Watson CTM had a median processing time of 15.5 seconds per case (range 7.2‐37.8 s) and achieved 97% agreement with human review [40]. Nevertheless, the workflow required 8088 manual actions to enter data or obtain clinician interpretation for eligibility, and Watson CTM did not query for missing information during the automated workflow. Reported accuracies for CTMs reflect performance largely on cases with sufficient information already available because the data-incomplete cases are excluded from the accuracy denominator [38].

### Implications

This proof-of-concept study holds several implications for the use of semiautonomous systems in real-world eligibility determinations. This type of workflow may address enrollment inefficiencies because prescreening and formal enrollment screening are interdependent. Inaccurate prescreening advances potential study participants who subsequently fail to enroll due to the more detailed formal screening process [27]. McKane et al [16] reported a 24.6% screen-failure rate in phase I trials, which rose 3-fold to 78.2% at a center that included inaccurate prescreening classification as part of the overall screen-failure rate. These failed screening efforts are also costly ($25,000 per patient) and resource-intensive (up to 8.5 h of staff time per patient) [41,42]. Overall, phase I trials commonly require accrual times 5-fold longer than originally planned because of the logistical obstacles created by complex eligibility criteria, such as biomarker and biopsy data [5,43,44]. Automated workflows, such as TrialTriage, may help to reduce costs and resource time by classifying cases accurately and quickly and by resolving ambiguous cases through an immediate email query to the investigator, although these benefits remain to be confirmed with real clinical data.

Bias can enter TrialTriage at the points where LLMs are used: extracting variables from the case, judging whether an investigator’s reply is substantive, and parsing substantive reply text into the data fields read by the rule engine. Several features of the architecture guard against errors at these points. Eligibility decisions are made by a deterministic rule engine applying threshold values, so the model does not rely on its own judgment. Investigator replies pass through a substantiveness check, and noninformative replies trigger an email recommending manual prescreening. The eligibility output is sent to the PI or research staff for final sign-off, and a timestamped audit trail records all classifications, queries, replies, and reclassifications. To prevent contamination of the EHR, TrialTriage reads only the source record and never writes back to it.

Some subjectivity and bias are likely to remain because some eligibility parameters, such as ECOG performance status (a graded measure of a patient’s ability to carry out daily activities), rely strongly on clinical judgment and cannot be classified by objective measures alone.

### Limitations

This study has several limitations, including the use of synthetic rather than real clinical cases, the limited independence of the test cases from the rules used to build the rule engine, small sample sizes, the limited set of eligibility criteria tested, and the absence of real-world data governance.

#### Synthetic Cases

The evaluation used synthetic case narratives rather than EHR-derived records. Synthetic cases are useful for testing workflow logic and rule implementation; however, because of their inherent simplicity, they cannot exactly reproduce the semantic and syntactic complexity of unstructured, real clinical documentation [38,45]. Actual medical records often contain inconsistencies, redundancies, conflicting documentation, and highly variable narrative quality, all of which complicate the extraction of clinically meaningful information [46-49]. Whether TrialTriage’s performance on synthetic cases would be maintained on real-world EHR narratives remains untested. Because the synthetic cases were not designed to reflect any patient demographic mix, the evaluation also cannot demonstrate how TrialTriage performs across different patient groups.

#### Test-Case Independence

The Version 2 test cases generated by Claude, Gemini, and Grok were built from variations on the same 7 eligibility criteria used to construct the rule engine during Version 1 development. The use of 3 different LLMs broadened the diversity of how cases were described but did not create a fully independent evaluation set. Performance against an external standard independent of the rule-based development process remains to be established.

#### Sample Size

The ambiguity-resolution analysis was limited to 6 randomly selected ambiguous cases from the Claude dataset, with the author responding to the system’s email queries as a simulated PI. The workflow handled those cases correctly; however, the sample is too small to support strong conclusions about how the system would perform with real investigator replies that vary in clarity, completeness, and timing. Whether real PIs or qualified study personnel would respond quickly enough to preserve the workflow’s time advantage remains untested. The reviewer comparison involved 5 clinical reviewers evaluating one 30-case dataset. That sample was sufficient to reveal general error patterns, particularly overinclusion and premature classification of ambiguous cases as eligible or not eligible, but it was not large enough to establish stable benchmarks for manual prescreening performance or to identify which case features most reliably produce reviewer error. The reviewer comparison should therefore be understood as hypothesis-generating rather than definitive.

#### Data Governance

Because the study used only synthetic cases and deliberately did not include protected health information, it cannot show how actual patient data would be governed within the workflow. Before using a semiautonomous system like TrialTriage in a phase I clinical trial, procedures for handling uploaded patient data must be established to ensure confidentiality. These procedures would specify where identifiable patient data enter the workflow, which steps process or store data, how long data are retained, how data are encrypted in storage and in transit, and how the investigator email channel is protected.

#### Criteria Scope

The workflow was tested on 7 representative criteria rather than the full complexity of a phase I protocol. Formal trial screening commonly involves more criteria, including biomarker requirements, disease extent, prior treatment history, organ function, and many other features derived from physical examination, laboratory data, and imaging. Typical phase I oncology protocols have upward of 60 eligibility criteria that must be met during formal screening [40]. Whether the same approach remains effective as criterion count and complexity expand will require further study.

### Future Directions

This proof-of-concept evaluation leaves 3 questions about the TrialTriage architecture unresolved, which could form the basis for future development.

#### Extraction Layer

The next stage of development would test TrialTriage on EHR-derived cases, with the goals of characterizing extraction performance on clinician-generated text and determining whether immediate, iterative email communication can shorten the time to final prescreening classification under real-world conditions. Operational end points for such testing include time to final prescreening decision, frequency of ambiguity resolution within a defined time window after the first email request, percentage of workload shifted away from manual review, prescreening failure rate (the number of patients referred for formal screening who were subsequently rejected), and screen-failure rate (the percentage of consented patients who were never dosed).

#### Human Factors

The performance of the human in the loop should be measured by the time taken to respond to the iterative emails and by the quality of the data supplied in reply. Implementations could direct email not only to the PI but also to designated research staff or coordinators, allowing a comparison of which responders provide the greatest gain in efficiency. TrialTriage must ultimately be tested in its integrated form, combining the semiautonomous classification workflow and the human-in-the-loop email response, to determine the overall accuracy and timing of the complete system.

#### Scalability

How the rule engine behaves as eligibility criteria increase, perhaps by a factor of 10 or more, is unknown. Testing performance with substantially more eligibility criteria must also include the human responders, whose replies are essential to the iterative process.

### Conclusions

TrialTriage demonstrated the feasibility of resolving prescreening ambiguity through immediate investigator email communication and iterative reclassification within the same automated workflow. If validated on EHR-derived cases and complex phase I trial criteria, the TrialTriage architecture could reduce manual prescreening workload and improve prescreening accuracy in early-phase oncology trials.

## Supplementary material

10.2196/100779 Multimedia Appendix 1 Screenshots of the deployed TrialTriage n8n workflow, showing the complete workflow canvas and detailed views of each functional section.

10.2196/100779 Multimedia Appendix 2 Table of n8n node types used in the TrialTriage workflow, with the function and number of deployed instances of each.

10.2196/100779 Multimedia Appendix 3 Prompt template used for generation of the synthetic pancreatic cancer case set across the 3 large language models reported in this study (Claude Sonnet 4.6, Gemini 3.1, and Grok 4).

10.2196/100779 Multimedia Appendix 4 Datasets generated and analyzed during the study, organized by source (large language model or human reviewer). Each tab contains the synthetic case classifications, the ground truth, and the corresponding TrialTriage workflow outputs.

10.2196/100779 Multimedia Appendix 5 Survey instrument used to collect case classifications and completion times from human reviewers for comparison against the TrialTriage workflow.

10.2196/100779 Checklist 1 TRIPOD-LLM checklist.

## Abbreviations

CTM: clinical trial matching
ECOG: Eastern Cooperative Oncology Group
EHR: electronic health record
LLM: large language model
PI: principal investigator
