# Supplementary material for: TrialTriage, a Semiautonomous Prescreening Workflow for Resolving Ambiguity in Phase I Oncology Trial Eligibility: Development and Proof-of-Concept Study Using Synthetic Cases
Source: JMIR Form Res. 2026 Aug 5;10:e100779. doi: 10.2196/100779 (PMC13440223; doi:10.2196/100779)
Supplement: Supplementary file 1 [file formative-v10-e100779-s001.docx]

**n8n workflow screenshots**

This appendix presents the deployed TrialTriage workflow as implemented in n8n. The workflow comprises 45 deployed node instances drawn from 20 unique node types, organized into five functional sections. An overview of the complete canvas is shown first, followed by detailed views of each section. Section labels visible at the bottom of each region group nodes by logical role: input file processing, criterion extraction and case categorization, first-pass investigator outreach, follow-up sequence for ambiguous cases, and final reclassification. Refer to Multimedia Appendix 2 for a tabular description of each node type and its function in the workflow.

 **Appendix 1: Figure 1.** Complete TrialTriage n8n workflow as deployed. Individual node labels may not be readable at this zoom level; detailed views of each section follow.

**Appendix 1: Figure 2.** Workflow Part 1: Processing input files from email. The Gmail Trigger detects new patient case files. The Switch node routes the workflow to one of four branches based on file format (XLSX, CSV, DOCX, or fallback). DOCX files are converted to text via the ConvertAPI HTTP Request. All formats converge at the Set Inputs node, which prepares data for the Information Extractor. Format errors are flagged by an If node that triggers an automated correction email to the principal investigator.

 **Appendix 1: Figure 3.** Workflow Part 2: Categorization (with evaluation pipeline). The Information Extractor (paired with the Anthropic Chat Model) extracts the seven eligibility criteria from each case narrative. The Code node executes deterministic rule-engine classification, producing Eligible (E), Not Eligible (NE), or Ambiguous (A) labels. The evaluation branch (top right) writes outputs to the evaluation dataset and computes performance metrics for benchmarking runs. The Switch routes classified cases to the appropriate downstream branch.

**Appendix 1: Figure 4.** Workflow Part 3: First emails to investigator. Eligible (E) and Not Eligible (NE) case lists are summarized and dispatched to the principal investigator immediately. Ambiguous (A) cases are routed to a separate branch that prepares a clarification email and sends it via the Send and Wait for Approval node, which pauses the workflow pending PI reply.

**Appendix 1: Figure 5.** Workflow Part 4: Follow-up sequence for ambiguous cases. If the PI does not respond to the first clarification request within 24 hours, a second request is sent. After the second request, the workflow either receives a reply (proceeding to substantiveness validation) or sends a final adjudication message recommending complete manual review. The Text Classifier (paired with an Anthropic Chat Model) evaluates whether a received reply contains substantive clinical information.

 **Appendix 1: Figure 6.** Workflow Part 5: Reclassification of ambiguous cases after PI reply. The AI Agent (paired with an Anthropic Chat Model and Structured Output Parser) extracts structured clinical information from the PI's free-text reply. The Split Out node decomposes the case array into individual cases, which are then routed by the Final Switch to one of three final classifications (Eligible, Not Eligible, or Ambiguous). Each branch produces a summary email delivered to the principal investigator.
