# Supplementary material for: TrialTriage, a Semiautonomous Prescreening Workflow for Resolving Ambiguity in Phase I Oncology Trial Eligibility: Development and Proof-of-Concept Study Using Synthetic Cases
Source: JMIR Form Res. 2026 Aug 5;10:e100779. doi: 10.2196/100779 (PMC13440223; doi:10.2196/100779)
Supplement: Supplementary file 2 [file formative-v10-e100779-s002.docx]

**n8n nodes used in the TrialTriage workflow**

The deployed workflow comprises 45 node instances drawn from 20 unique node types. Some node types appear in multiple instances because the workflow has three parallel branches for the three classification categories (Eligible, Not Eligible, Ambiguous) and an iterative, follow-up sequence that sends a second email if the principal investigator does not reply within 24 hours.

| **Node Type** | **Function in TrialTriage Workflow** | **Instances** |
| --- | --- | --- |
| Gmail Trigger | Detects new email containing patient case file and initiates the workflow. | 1 |
| Switch | Routes incoming items to the appropriate file-format branch (XLSX, CSV, or DOCX) and routes classified cases to E, NE, or A branches. | 3 |
| HTTP Request | Calls ConvertAPI to convert DOCX attachments to Base64-encoded text for downstream parsing. | 1 |
| Extract from File (XLSX) | Parses XLSX attachments into structured rows for case-by-case processing. | 1 |
| Extract from File (CSV) | Parses CSV attachments into structured rows for case-by-case processing. | 1 |
| Code (JavaScript) | Executes deterministic rule-engine logic for criterion-by-criterion classification and reclassification of ambiguous cases after PI reply. | 4 |
| Edit Fields (Set) | Reshapes data structures between processing stages and adds case identifiers. | 3 |
| If | Branches the workflow based on file-format validity, response substantiveness, or PI reply receipt. | 4 |
| Information Extractor | Uses an LLM to extract values for the seven eligibility criteria from unstructured case narratives. | 1 |
| Text Classifier | Uses an LLM to classify a PI reply as substantive or non-substantive. | 1 |
| Anthropic Chat Model | Provides Claude Sonnet 4.6 as the underlying chat model attached to the Information Extractor, Text Classifier, and AI Agent nodes. | 3 |
| AI Agent | Validates whether a PI reply contains substantive clinical information sufficient to support reclassification of an ambiguous case. | 1 |
| Structured Output Parser | Constrains LLM output to a defined JSON schema for predictable downstream parsing. | 1 |
| Split Out | Decomposes case arrays into individual case items for per-case routing and classification. | 1 |
| Summarize | Aggregates classified cases into per-category lists (E, NE, A) for delivery to the principal investigator. | 5 |
| Gmail (Send a message) | Sends classified case lists, format-correction requests, follow-up clarifications, and final adjudication messages to the principal investigator. | 9 |
| Gmail (Send and Wait for Approval) | Sends initial and follow-up ambiguous-case clarification requests and pauses the workflow pending PI reply. | 2 |
| Evaluation Trigger | Pulls one row at a time from the evaluation dataset to feed the workflow during benchmarking. | 1 |
| Evaluation (setMetrics) | Records numeric performance metrics for each evaluation row to support model scoring. | 1 |
| Evaluation (setOutputs) | Writes model outputs back to the evaluation dataset for comparison against expected answers. | 1 |
| **Total deployed instances** | | **45** |
