# Supplementary material for: TrialTriage, a Semiautonomous Prescreening Workflow for Resolving Ambiguity in Phase I Oncology Trial Eligibility: Development and Proof-of-Concept Study Using Synthetic Cases
Source: JMIR Form Res. 2026 Aug 5;10:e100779. doi: 10.2196/100779 (PMC13440223; doi:10.2196/100779)
Supplement: Supplementary file 3 [file formative-v10-e100779-s003.docx]

**Prompt template for synthetic case generation**

The following prompt was used identically across all three generating models: Claude Sonnet 4.6 (Anthropic), Gemini-3.1 (Google), and Grok 4 (xAI).

**Prompt:**

Generate 30 synthetic cases of pancreatic cancer for evaluation of a pre-screening eligibility classification system. Each case should describe a patient being considered for a theoretical Phase I clinical trial, using 2 to 3 sentences that include the patient's age, diagnosis, ECOG performance status, and laboratory values for SGOT (AST), SGPT (ALT), total bilirubin, and creatinine.

The seven eligibility criteria are: (1) pancreatic adenocarcinoma confirmed by pathology or biopsy within the prior 6 months; (2) age 18 to 80 years; (3) ECOG performance status 0 or 1; (4) SGOT within protocol limits (40 U/L or less); (5) SGPT within protocol limits (40 U/L or less); (6) total bilirubin within protocol limits (1.2 mg/dL or less); (7) creatinine within protocol limits (1.5 mg/dL or less).

Of the 30 cases, 10 should be Eligible (E), meaning all 7 criteria are explicitly met with values within protocol limits. Ten should be Not Eligible (NE), meaning one or more criteria explicitly fails because a laboratory value is out of range, age is not met or exceeded, ECOG is too high, or the diagnosis window is exceeded. Ten should be Ambiguous (A), meaning one or more criteria is missing, illegible, or stated in a form that cannot be definitively evaluated (for example, a pathology date is not specified, ECOG is described functionally rather than numerically such as "patient appears well," or a laboratory value is absent from the submitted panel).

Each case must be original and independently generated. Output three columns: Case ID, Case Narrative, and Classification (E, NE, or A).
