# Supplementary material for: TrialTriage, a Semiautonomous Prescreening Workflow for Resolving Ambiguity in Phase I Oncology Trial Eligibility: Development and Proof-of-Concept Study Using Synthetic Cases
Source: JMIR Form Res. 2026 Aug 5;10:e100779. doi: 10.2196/100779 (PMC13440223; doi:10.2196/100779)
Supplement: Supplementary file 4 [file formative-v10-e100779-s005.docx]

# TrialTriage Case Classification - Timed Exercise

**Name____________________ Date________________**

## Purpose

(1) This exercise measures the time and accuracy for humans to perform pre-screening based on artificial medical records for a theoretical trial involving pancreatic cancer.

(2) You will review 30 synthetic (artificial) cases for enrollment eligibility after reviewing 7 inclusion and exclusion criteria.

(3) Your task is to read the few sentences describing each synthetic case and decide if the patient is eligible for trial entry (E), not eligible for entry (NE), or ambiguous (A), meaning you are not certain if the patient is eligible or ineligible because of missing or unclear information.

(4) To classify, please circle E, NE, or A on the sheet, just after the case number.

(5) The time it takes for you to classify these cases will be determined from Finish Time - Start Time (in minutes).

## Instructions

**1. Record your start time before reading Case 1.**

**START TIME:____________**

2. Review each case and classify it using exactly one of three labels shown below.

3. Classify each case by circling either E / NE / A for each case as you go. Please do not revisit prior cases

| **E** | **Eligible** | All 7 inclusion criteria explicitly met with values within normal limits |
| --- | --- | --- |
| **NE** | **Not Eligible** | One or more criteria explicitly fails because lab value out of range, age not met or exceeded, ECOG too high, or diagnosis window exceeded |
| **A** | **Ambiguous** | One or more criteria is missing, illegible, or stated in a form that cannot be definitively evaluated |

3. Work through all 30 cases without revisiting prior cases.

4. Record your end time after completing Case 30.

5. Calculate: Total time (minutes) and your classification for each case.

## Eligibility Criteria — All 7 Must Be Met for Patient to be Eligible

| **#** | **Criterion** |
| --- | --- |
| **1** | Pancreatic adenocarcinoma confirmed by pathology or biopsy within the prior 6 months |
| **2** | Age 18–80 years |
| **3** | ECOG performance status 0 or 1 |
| **4** | SGOT (AST) within normal limits (≤ 40 U/L) |
| **5** | SGPT (ALT) within normal limits (≤ 40 U/L) |
| **6** | Total bilirubin within normal limits (≤ 1.2 mg/dL) |
| **7** | Creatinine within normal limits (≤ 1.5 mg/dL) |

**Normal limits applied:** SGOT and SGPT ≤ 40 U/L | Total bilirubin ≤ 1.2 mg/dL | Creatinine ≤ 1.5 mg/dL

# Patient Cases

**Case 1: E......NE......A**

A 67-year-old man with pancreatic adenocarcinoma confirmed by core biopsy 3 months ago is referred for trial screening. ECOG performance status is 1. Laboratory studies show SGOT 32 U/L, SGPT 34 U/L, total bilirubin 0.9 mg/dL, and creatinine 1.1 mg/dL. He has hypertension managed with lisinopril and reports no new symptoms.

**Case 2: E......NE......A**

A 71-year-old woman with pancreatic adenocarcinoma confirmed by pathology 2 months ago presents for eligibility review. ECOG performance status is 2. SGOT is 28 U/L, SGPT is 30 U/L, total bilirubin is 0.8 mg/dL, and creatinine is 1.0 mg/dL. She reports significant fatigue limiting daily activities and uses a cane for ambulation.

**Case 3: E......NE......A**

A 58-year-old man with biopsy-confirmed pancreatic cancer diagnosed 4 months ago is evaluated for study entry. ECOG performance status is not recorded in the current oncology note. SGOT is 26 U/L, SGPT is 29 U/L, total bilirubin is 0.7 mg/dL, and creatinine is 0.9 mg/dL. He takes omeprazole for GERD and has no other active medical problems.

**Case 4: E......NE......A**

A 54-year-old woman with pancreatic adenocarcinoma confirmed on pathology 1 month ago presents for screening. ECOG performance status is 0. SGOT is 24 U/L, SGPT is 27 U/L, total bilirubin is 0.6 mg/dL, and creatinine is 0.8 mg/dL. She has no significant comorbidities and remains fully active, working full-time.

**Case 5: E......NE......A**

An 83-year-old man with pancreatic adenocarcinoma confirmed by biopsy 2 months ago is considered for enrollment. ECOG performance status is 1. SGOT is 30 U/L, SGPT is 28 U/L, total bilirubin is 0.8 mg/dL, and creatinine is 1.0 mg/dL. He has mild hearing loss and paroxysmal atrial fibrillation managed with a beta-blocker.

**Case 6: E......NE......A**

A 62-year-old woman with pancreatic cancer confirmed by pathology 3 months ago is referred for trial consideration. ECOG performance status is 1. SGOT is 27 U/L, SGPT is 31 U/L, and creatinine is 1.0 mg/dL. Total bilirubin is not included in the laboratory panel submitted with the referral. She has type 2 diabetes managed with metformin.

**Case 7: E......NE......A**

A 75-year-old man with biopsy-confirmed pancreatic adenocarcinoma diagnosed 5 months ago is assessed for the study. ECOG performance status is 1. SGOT is 35 U/L, SGPT is 37 U/L, total bilirubin is 1.1 mg/dL, and creatinine is 1.3 mg/dL. He has stable chronic obstructive pulmonary disease managed with an inhaled bronchodilator and remains ambulatory.

**Case 8: E......NE......A**

A 66-year-old woman with pancreatic adenocarcinoma confirmed by biopsy 2 months ago is screened for enrollment. ECOG performance status is 0. SGOT is 68 U/L, SGPT is 38 U/L, total bilirubin is 0.9 mg/dL, and creatinine is 1.0 mg/dL. She has a history of non-alcoholic fatty liver disease and reports no current alcohol use.

**Case 9: E......NE......A**

A 70-year-old man with pancreatic adenocarcinoma confirmed by core biopsy is evaluated for trial entry. The pathology report date in the available chart records is not legible. ECOG performance status is 1. SGOT is 29 U/L, SGPT is 31 U/L, total bilirubin is 0.8 mg/dL, and creatinine is 1.0 mg/dL. He has well-controlled hypertension on amlodipine.

**Case 10: E......NE......A**

A 61-year-old woman with pancreatic cancer confirmed by pathology 3 months ago presents for review. ECOG performance status is 1. SGOT is 28 U/L, SGPT is 30 U/L, total bilirubin is 0.9 mg/dL, and creatinine is 2.3 mg/dL. She carries a diagnosis of stage 3 chronic kidney disease and has mild peripheral edema.

**Case 11: E......NE......A**

A 48-year-old man with pancreatic adenocarcinoma confirmed by biopsy 2 months ago is referred for screening. ECOG performance status is 0. SGOT is 22 U/L, SGPT is 25 U/L, total bilirubin is 0.5 mg/dL, and creatinine is 0.9 mg/dL. He has mild seasonal allergies treated with cetirizine as needed and is otherwise healthy and physically active.

**Case 12: E......NE......A**

A 59-year-old man with pancreatic adenocarcinoma confirmed by pathology 1 month ago is evaluated for study eligibility. ECOG performance status is 1. SGOT is 33 U/L, SGPT is 36 U/L, total bilirubin is 4.1 mg/dL, and creatinine is 1.0 mg/dL. He has progressive obstructive jaundice; biliary stenting is being planned.

**Case 13: E......NE......A**

A 64-year-old woman with pancreatic cancer confirmed by biopsy 4 months ago is considered for enrollment. ECOG performance status is 0. SGOT is 25 U/L, SGPT is 28 U/L, and total bilirubin is 0.7 mg/dL. Creatinine is absent from the laboratory panel submitted with the referral documents. She has hypothyroidism managed with levothyroxine.

**Case 14: E......NE......A**

A 69-year-old man with biopsy-confirmed pancreatic adenocarcinoma diagnosed 3 months ago is screened for the study. ECOG performance status is 1. SGOT is 31 U/L, SGPT is 33 U/L, total bilirubin is 1.0 mg/dL, and creatinine is 1.2 mg/dL. He has benign prostatic hyperplasia on tamsulosin and controlled hyperlipidemia on rosuvastatin.

**Case 15: E......NE......A**

A 57-year-old woman with pancreatic adenocarcinoma confirmed by pathology 8 months ago is referred for trial screening. ECOG performance status is 0. SGOT is 24 U/L, SGPT is 26 U/L, total bilirubin is 0.8 mg/dL, and creatinine is 0.9 mg/dL. She has migraines treated with sumatriptan as needed and reports no other active medical conditions.

**Case 16: E......NE......A**

A 73-year-old man with pancreatic cancer confirmed by biopsy 2 months ago is assessed for the study. ECOG performance status is documented as "mildly symptomatic with some limitation in strenuous activity" with no numeric score recorded in the chart. SGOT is 28 U/L, SGPT is 30 U/L, total bilirubin is 0.8 mg/dL, and creatinine is 1.0 mg/dL. He has controlled hypertension on amlodipine.

**Case 17: E......NE......A**

A 52-year-old woman with pancreatic adenocarcinoma confirmed by pathology 5 months ago is evaluated for study entry. ECOG performance status is 0. SGOT is 23 U/L, SGPT is 25 U/L, total bilirubin is 0.7 mg/dL, and creatinine is 0.8 mg/dL. She takes sertraline for mild anxiety and has no other daily prescription medications.

**Case 18: E......NE......A**

A 65-year-old man with biopsy-confirmed pancreatic adenocarcinoma diagnosed 2 months ago is screened for enrollment. ECOG performance status is 1. SGOT is 38 U/L, SGPT is 57 U/L, total bilirubin is 1.0 mg/dL, and creatinine is 1.1 mg/dL. He has a history of alcohol use disorder; he reports complete abstinence for two years. Liver imaging shows hepatic steatosis.

**Case 19: E......NE......A**

A 60-year-old woman with pancreatic cancer confirmed on pathology 3 months ago presents for review. ECOG performance status is 1. SGPT is 32 U/L, total bilirubin is 0.8 mg/dL, and creatinine is 1.0 mg/dL. The SGOT result is not present in the submitted laboratory panel. She has hyperlipidemia on atorvastatin and occasional knee pain.

**Case 20: E......NE......A**

A 78-year-old woman with biopsy-confirmed pancreatic adenocarcinoma diagnosed 4 months ago is referred for screening. ECOG performance status is 1. SGOT is 33 U/L, SGPT is 36 U/L, total bilirubin is 0.9 mg/dL, and creatinine is 1.4 mg/dL. She has osteoporosis managed with calcium and vitamin D supplementation and remains independently ambulatory.

**Case 21: E......NE......A**

A 68-year-old man with pancreatic adenocarcinoma confirmed by pathology 1 month ago is assessed for trial eligibility. ECOG performance status is 3. SGOT is 30 U/L, SGPT is 32 U/L, total bilirubin is 0.8 mg/dL, and creatinine is 1.0 mg/dL. He requires significant assistance with self-care activities and spends the majority of daytime hours in bed or in a chair.

**Case 22: E......NE......A**

A 55-year-old woman with pancreatic cancer presents for screening. Per patient report, she was diagnosed "approximately 5 to 7 months ago." Outside pathology records have been requested but are not yet available in the chart. ECOG performance status is 0. SGOT is 25 U/L, SGPT is 27 U/L, total bilirubin is 0.7 mg/dL, and creatinine is 0.9 mg/dL. She has diet-controlled type 2 diabetes.

**Case 23: E......NE......A**

A 63-year-old man with pancreatic adenocarcinoma confirmed by core biopsy 2 months ago is evaluated for the study. ECOG performance status is 0. SGOT is 27 U/L, SGPT is 29 U/L, total bilirubin is 0.8 mg/dL, and creatinine is 1.0 mg/dL. He has controlled atrial fibrillation on apixaban and takes no other prescription medications.

**Case 24: E......NE......A**

A 72-year-old woman with pancreatic adenocarcinoma confirmed by biopsy 3 months ago is considered for enrollment. ECOG performance status is 1. SGOT is 92 U/L, SGPT is 105 U/L, total bilirubin is 1.0 mg/dL, and creatinine is 1.1 mg/dL. She has a prior history of biliary obstruction treated with endoscopic stenting; imaging shows no current biliary dilation.

**Case 25: E......NE......A**

A 67-year-old woman with pancreatic cancer confirmed by pathology 2 months ago is screened for the study. ECOG performance status is 1. SGOT is 29 U/L, SGPT is 31 U/L, and total bilirubin is 0.9 mg/dL. The clinic note records creatinine as "mildly elevated compared to prior baseline" with no numeric value provided. She has stage 2 chronic kidney disease.

**Case 26: E......NE......A**

A 57-year-old man with biopsy-confirmed pancreatic adenocarcinoma diagnosed 1 month ago is referred for trial consideration. ECOG performance status is 1. SGOT is 30 U/L, SGPT is 32 U/L, total bilirubin is 0.8 mg/dL, and creatinine is 1.1 mg/dL. He has controlled hyperlipidemia on rosuvastatin and reports no change in functional status.

**Case 27: E......NE......A**

A 64-year-old woman with pancreatic adenocarcinoma confirmed by pathology 9 months ago is assessed for enrollment. ECOG performance status is 1. SGOT is 26 U/L, SGPT is 28 U/L, total bilirubin is 0.8 mg/dL, and creatinine is 1.0 mg/dL. She has controlled hypothyroidism on levothyroxine and has been undergoing imaging follow-up at an outside institution.

**Case 28: E......NE......A**

A patient with pancreatic adenocarcinoma confirmed by biopsy 3 months ago is referred for screening. The patient's date of birth is recorded inconsistently across two chart entries: one lists 1943 (age approximately 82) and the other lists 1953 (age approximately 72). ECOG performance status is 1. SGOT is 28 U/L, SGPT is 30 U/L, total bilirubin is 0.8 mg/dL, and creatinine is 1.0 mg/dL. If the earlier date of birth is correct, the patient would exceed the upper age limit.

**Case 29: E......NE......A**

A 45-year-old man with pancreatic adenocarcinoma confirmed by pathology 2 months ago presents for trial screening. ECOG performance status is 0. SGOT is 21 U/L, SGPT is 24 U/L, total bilirubin is 0.6 mg/dL, and creatinine is 0.8 mg/dL. He has no significant past medical history, takes no prescription medications, and remains physically active.

**Case 30: E......NE......A**

A 71-year-old woman with pancreatic cancer confirmed by biopsy 4 months ago is evaluated for the study. ECOG performance status is 1. SGOT is 30 U/L, total bilirubin is 0.9 mg/dL, and creatinine is 1.0 mg/dL. SGPT is not available in the submitted laboratory panel. She has well-controlled type 2 diabetes on basal insulin and denies recent changes in functional status.

**End time: _______________**

**Time to complete: (End Time) - (Start Time) = Total time:** _______________ (minutes)
