# Supplementary material for: TrialTriage, a Semiautonomous Prescreening Workflow for Resolving Ambiguity in Phase I Oncology Trial Eligibility: Development and Proof-of-Concept Study Using Synthetic Cases
Source: JMIR Form Res. 2026 Aug 5;10:e100779. doi: 10.2196/100779 (PMC13440223; doi:10.2196/100779)
Supplement: Supplementary file 5 [file formative-v10-e100779-s006.docx]

# Completed TRIPOD-LLM Reporting Checklist

Manuscript #100779 — TrialTriage. Checklist from Gallifant J, et al. The TRIPOD-LLM reporting guideline for studies using large language models. Nat Med. 2025;31(1):60–69. doi:10.1038/s41591-024-03425-5.

*Applicable TRIPOD-LLM design categories for this study: LLM evaluation (E), classification task (C). The study uses existing released LLMs and does not involve de novo development (D), LLM methods development (M), fine-tuning, or generative tasks (QA, IR, DG, SS, MT). Items coded only for those designs or tasks are marked Not applicable with a brief reason, per the modular format of the guideline.*

*Each checklist item is mapped to where it is addressed in the manuscript. Items not applicable to this study design are marked accordingly with a brief reason.*

| **Item** | **Checklist item (abbreviated; see TRIPOD-LLM for full wording)** | **Reported? Location in manuscript / response** |
| --- | --- | --- |
| **Title** | | |
| **1** | Identify the study as developing, fine-tuning, and/or evaluating an LLM, specifying the task, target population, and outcome. | **Reported. Title and Abstract.**  The title identifies the study as the development and synthetic-case evaluation of TrialTriage, a semi-autonomous prescreening workflow, names the task (resolving eligibility ambiguity at prescreening), and the population (Phase I oncology trial candidates). |
| **Abstract** | | |
| **2** | Structured abstract per TRIPOD-LLM for Abstracts (background, objective, methods, results, conclusions). | **Reported. Abstract.**  Structured Background, Objective, Methods, Results, Conclusions. States the task, the synthetic dataset, the three LLMs, the rule engine, the human comparison, and the proof-of-concept framing. |
| **Introduction** | | |
| **3a** | Explain the healthcare context / use case and rationale for the LLM, with references to existing approaches and models. | **Reported. Introduction, paragraphs 1–7.**  Describes Phase I oncology accrual, prescreening, the bottleneck created when ambiguous cases are routed to offline manual review, and existing clinical trial matching systems, with references. |
| **3b** | Describe the target population and intended use within the care pathway, including intended users (code E,H). | **Reported. Introduction (final paragraphs) and Discussion (Principal Findings).**  Intended use is investigator-facing prescreening support; intended users are the principal investigator and research staff. The system operates downstream of candidate identification and before formal screening. |
| **4** | Specify study objectives, including whether the study is initial development, fine-tuning, or validation (or multiple stages). | **Reported. Introduction, final paragraph (revised).**  States the aim and three hypotheses, and clarifies this is development plus internal synthetic-case evaluation of an existing-LLM workflow, not training or fine-tuning. |
| **Methods — Data** | | |
| **5a** | Describe sources of data for training, tuning, and/or evaluation, and the rationale. | **Reported. Methods (Synthetic Dataset Generation; Evaluation Procedure).**  No training or tuning data were used; the embedded LLMs were used as released. Evaluation data were 90 synthetic narratives generated by Claude Sonnet 4.6, Gemini 3.1, and Grok 4. Rationale for synthetic data is stated (no PHI; controlled criteria coverage). |
| **5b** | Describe relevant data points and the distribution and descriptors of the dataset. | **Reported. Methods; Table 1; Multimedia Appendix 4.**  Seven eligibility variables defined in Table 1. Each model produced 10 eligible, 10 not eligible, and 10 ambiguous cases. Full case listings and labels are in Appendix 4. |
| **5c** | State the date of the oldest and newest text used in development and in the evaluation datasets. | **Reported. Methods (Synthetic Dataset Generation; Evaluation Procedure).**  The synthetic cases were generated in February 2026. TrialTriage classification of all 90 cases was performed in March 2026, and the five reviewers completed manual classification between March 4 and March 30, 2026. No human-authored text corpus was used. |
| **5d** | Describe data pre-processing and quality checking, including consistency across sources and sociodemographic groups. | **Reported. Methods (Ground Truth; Data Ingestion, Validation, and Initial Classification).**  Input validation checks file and data format. Answer keys were checked by the author against the criteria; the pancreatic cancer / adenocarcinoma synonym issue was identified and corrected. Cases were not constructed to represent any demographic distribution (stated in Bias, Oversight, and Assumptions). |
| **5e** | Describe how missing and imbalanced data were handled and reasons for omitting data. | **Reported. Methods (Eligibility Criteria; Investigator Clarification).**  Missing or non-numeric values for any criterion force an Ambiguous classification, which triggers the investigator email loop. Categories were balanced by design (10/10/10 per model). No cases were omitted from the 90-case evaluation. |
| **Methods — Analytical methods** | | |
| **6a** | Report the LLM name, version, and last date of training. | **Reported. Methods (System Architecture).**  The embedded model is Claude Sonnet 4.6 (Anthropic). Last training date is not publicly disclosed by the developer; stated as not available, per the TRIPOD-LLM note for studies using existing LLMs. |
| **6b** | Report LLM development process: architecture, training, fine-tuning, alignment strategy and goals (code M,D). | **Not applicable.**  *No de novo development, training, fine-tuning, or alignment was performed. Existing released models were used through the n8n platform. Item applies to development studies (M/D) only.* |
| **6c** | Report how text was generated: prompt engineering, output consistency, inference settings (seed, temperature, max tokens, penalties). | **Reported. Methods (System Architecture; Synthetic Dataset Generation); Multimedia Appendix 3.**  The three LLM-based node types and their roles are described. The case-generation prompt template is in Appendix 3. The embedded model was run through n8n's native Claude integration using the platform's default inference settings; no custom temperature, token limit, or penalty values were set. |
| **6d** | Specify the initial and post-processed output of the LLM (probabilities, classification, unstructured text). | **Reported. Methods (System Architecture; Closed-Loop Workflow Execution).**  LLM nodes output extracted structured variables and a substantive/nonsubstantive reply label. The final case classification (E/NE/A) is produced by the deterministic rule engine, not the LLM. |
| **6e** | Provide rationale for classification and, if applicable, how probabilities and thresholds were determined (code C, OF). | **Reported. Methods (Eligibility Criteria, Table 1; Classification Categories).**  Classification is rule-based, not probabilistic. The seven criteria and their numeric thresholds are specified in Table 1. A case is Eligible only if all seven criteria are met; missing or out-of-range values yield Not Eligible or Ambiguous. No probability thresholds are used. |
| **Methods — LLM output and outcome** | | |
| **7a** | Include metrics for quality of generative outputs (consistency, relevance, accuracy vs gold standards) (task codes QA/IR/DG/SS/MT). | **Not applicable as a generative task.**  *The workflow performs classification, not long-form generation, question answering, summarization, or translation. Classification accuracy against ground truth is reported under item 17. Extraction reliability is reflected in 100% concordance on prompt-faithful cases.* |
| **7b** | Report relevance of outcome metrics to the downstream deployment task and correlation with human evaluation (code E,H). | **Reported. Methods (Statistical Analysis); Results; Discussion.**  The downstream task is correct prescreening classification and correct routing of ambiguous cases. Performance is compared directly to five human reviewers on the same cases. |
| **7c** | Define the outcome, how predictions were calculated (formula, code, API), inference date for closed-source LLMs, and metrics (code E,H). | **Reported. Methods (Statistical Analysis; Evaluation Procedure).**  Outcome is the E/NE/A label. Predictions are computed by the rule engine from LLM-extracted variables. Metrics are accuracy with exact binomial CI, Cohen kappa, and Fleiss kappa. Inference dates added per item 5c. |
| **7d** | If outcome assessment requires subjective interpretation, describe assessor qualifications, instructions, demographics, and inter-assessor agreement. | **Reported. Methods (Comparison of Manual Prescreening Performance to TrialTriage); Results (Table 2).**  Five reviewers (three physicians, two nonphysician clinicians, all with drug-development experience) classified the Claude dataset using uniform written instructions. Inter-rater agreement is reported as Fleiss kappa = 0.910. No personal demographic data beyond professional role were collected (stated in Ethical Considerations). |
| **7e** | Specify how performance was compared to other LLMs, humans, and benchmarks. | **Reported. Methods; Results (Tables 2–3); Discussion (Comparison With Prior Studies).**  Compared to five human reviewers on accuracy and time, across three LLM-generated datasets for cross-model consistency, and against published matching systems in the Discussion. |
| **Methods — Annotation** | | |
| **8a** | If annotation was done, report how text was labeled, with guidelines and examples. | **Reported. Methods (Synthetic Dataset Generation; Ground Truth); Multimedia Appendix 5.**  Ground-truth labels were produced by each generating model and then verified by the author against the seven criteria. Human reviewers received written classification instructions (Appendix 5). |
| **8b** | Report number of annotators, proportion double-annotated, and inter-annotator agreement. | **Reported. Methods (Comparison subsection); Results (Fleiss kappa).**  Five reviewers each independently classified all 30 Claude cases (100% overlap). Inter-rater agreement Fleiss kappa = 0.910 (95% CI 0.813–0.980). |
| **8c** | Provide background and experience of annotators or characteristics of labeling models. | **Reported. Methods (Comparison subsection).**  Reviewer roles and drug-development experience are described. The generating models (Claude, Gemini, Grok) and their versions are named. |
| **Methods — Prompting** | | |
| **9a** | If prompting was used, provide details on prompt design, curation, and selection. | **Reported. Methods (System Architecture; Synthetic Dataset Generation); Multimedia Appendix 3.**  The case-generation prompt template is provided in Appendix 3. The extraction and reply-classification node prompts are described in Methods. |
| **9b** | If prompting was used, report what data were used to develop the prompts. | **Reported. Methods.**  Prompts were built from the seven protocol criteria in Table 1, not from any external patient data. |
| **Methods — Task-specific items (not applicable to this study)** | | |
| **10** | Summarization: describe preprocessing before summarization (task code SS). | **Not applicable.**  *The workflow does not perform summarization.* |
| **11** | Instruction tuning/alignment: instructions, data, interface, and evaluation populations (code M,D). | **Not applicable.**  *No instruction tuning or alignment was performed; existing models were used as released.* |
| **12** | Compute required to carry out methods (machines, cost, inference time, FLOPs) (code M,D,E). | **Partially reported.**  *Wall-clock processing time is reported (1.8–2.8 minutes per 30-case dataset; Results, System Processing Time). Hardware, cost, and FLOPs are not applicable, since inference ran on the vendor-hosted model through n8n with no local compute provisioned by the author.* |
| **Methods — Ethical approval** | | |
| **13** | Name the IRB/ethics committee that approved the study and describe informed consent or the waiver. | **Reported. Methods (Ethical Considerations, new subsection).**  The study did not involve human subjects or real patient data and did not meet the regulatory definition of human subjects research (45 CFR 46.102); IRB review and informed consent were not applicable, and no approval or exemption was sought because none was required. The five reviewers participated as expert colleagues in a methods comparison, not as research participants. |
| **Open science** | | |
| **14a** | Source of funding and role of funders. | **Reported. Funding statement.**  The author declared that no financial support was received for this work. |
| **14b** | Conflicts of interest and financial disclosures for all authors. | **Reported. Conflicts of Interest statement.**  None declared. |
| **14c** | Where the study protocol can be accessed, or state none prepared (code H). | **Reported.**  No separate formal protocol was prepared; the methods are fully described in the manuscript. The eligibility criteria and workflow specification serve as the operational protocol. |
| **14d** | Study registration information, or state not registered (code H). | **Reported.**  The study was not registered. It is a methods development and evaluation study using synthetic data, not a clinical trial, and does not fall under trial-registration requirements. |
| **14e** | Availability of the study data. | **Reported. Data Availability Statement; Multimedia Appendix 4.**  All synthetic case data, ground-truth labels, and workflow outputs are provided as Multimedia Appendices. |
| **14f** | Availability of the code to reproduce results. | **Reported. Methods; Multimedia Appendices 1–2.**  The n8n workflow architecture is documented in Appendices 1–2 (node screenshots and node-type listing). The Python code used for the kappa and bootstrap calculations (Python 3.12.3 with NumPy 2.4.4) is available from the author on request. |
| **Patient and public involvement** | | |
| **15** | Details of any patient and public involvement, or state none (code H). | **Reported.**  There was no patient or public involvement. The study used synthetic cases and no real patients or members of the public participated. |
| **Results — Participants** | | |
| **16a** | When using patient/EHR data, describe flow of data through the study (code E,H). | **Not applicable (no patient/EHR data).**  *No patient or EHR data were used. The flow of the 90 synthetic cases through classification and the email loop is described in Results and Figure 1.* |
| **16b** | When using patient/EHR data, report characteristics by source and by development/evaluation split (code E,H). | **Not applicable (no patient/EHR data).**  *Synthetic dataset composition (10/10/10 per model across three models) is reported in Methods and Appendix 4.* |
| **16c** | For clinical-outcome evaluation, compare distribution of clinical variables between development and evaluation data (code E,H). | **Not applicable.**  *No clinical outcomes and no separate development/evaluation patient cohorts; the evaluation used synthetic cases only.* |
| **16d** | When using patient/EHR data, specify participants and outcome events in each analysis (code E,H). | **Not applicable (no patient/EHR data).**  *Case counts per analysis (90 total; 30 per model; 6 ambiguous cases in the email-loop subset) are reported in Results.* |
| **Results — Performance and updating** | | |
| **17** | Report LLM performance per pre-specified metrics and/or human evaluation. | **Reported. Results (TrialTriage Classification Performance; Manual Reviewer Performance; Tables 2–3).**  100% concordance with ground truth across 90 cases (95% CI 96.0–100.0%); processing time 1.8–2.8 min per 30 cases; reviewer accuracy mean 96.7% with Fleiss kappa 0.910. |
| **18** | If applicable, report results of any LLM updating and subsequent performance. | **Not applicable.**  *The LLMs were not updated during the study. The deterministic rule engine was corrected once to treat “pancreatic cancer” and “pancreatic adenocarcinoma” as equivalent; this rule-engine change is reported in Methods (Ground Truth).* |
| **Discussion** | | |
| **19a** | Overall interpretation of results, including fairness, in context of objectives and prior studies. | **Reported. Discussion (Principal Findings; Comparison With Prior Studies; Bias, Oversight, and Assumptions).**  Interprets the concordance and time findings, contrasts with prior matching systems, and addresses fairness and subgroup performance in the new bias subsection. |
| **19b** | Limitations and their effects on bias, statistical uncertainty, and generalizability. | **Reported. Discussion (Limitations).**  Covers synthetic data, non-independent evaluation set, small ambiguity-loop sample, single simulated responder, small reviewer panel, and reduced criterion count. |
| **19c** | Known challenges in using data for the task and domain: representation, missingness, harmonization, bias (code E,H). | **Reported. Discussion (Limitations; Bias, Oversight, and Assumptions).**  Discusses real-record complexity, missing and conflicting documentation, and the synonym harmonization issue, and the inability of synthetic data to characterize subgroup performance. |
| **19d** | Define intended use: intended input, end-user, level of autonomy/human oversight (code E,H). | **Reported. Discussion (Principal Findings; Bias, Oversight, and Assumptions); Methods.**  Semi-autonomous by design; the final eligibility decision remains with the PI. Input is case narratives; end-users are the PI and research staff; the rule engine makes the deterministic classification and all output is PI-reviewed. |
| **19e** | How poor-quality or unavailable input should be assessed and handled at implementation (code E,H). | **Reported. Methods (Investigator Clarification); Discussion.**  Missing or unclear data force an Ambiguous label and trigger the investigator email loop; unresolved cases after the third email are released to manual review. Input validation rejects malformed files. |
| **19f** | Whether users must interact in handling input or use of the LLM, and required expertise (code E,H). | **Reported. Methods; Discussion (Future Directions).**  Users interact by replying to clarification emails with specific clinical values; the required expertise is that of a PI, coordinator, or research clinician familiar with the case. |
| **19g** | Next steps for future research, with a view to applicability and generalizability. | **Reported. Discussion (Future Directions).**  Testing on EHR-derived cases, operational endpoints, escalation to named secondary reviewers, a real-time dashboard, and subgroup performance evaluation. |

*Page numbers are intentionally referenced by section rather than fixed page, because the revised manuscript pagination will change after track changes are accepted. Replace section references with final page numbers if the journal requests them.*
